# January 23: A date for COVID-19 research and reflection

**DOI:** 10.7189/jogh.13.03056

**Published:** 2023-09-29

**Authors:** Jie Huang, Weiliang Tian, Ole Döring

**Affiliations:** 1 School of Public Health and Emergency Management, Southern University of Science and Technology, Shenzhen, Guangdong Province, China; 2 Institute for Global Health and Development, Peking University, Beijing, China; 3 Department of Global Statistics, Eli Lilly and Company, Branchburg, New Jersey, USA; 4 School of Foreign Language Studies, Hunan Normal University, Changsha, China; 5 Institute for Technology Futures, Karlsruhe Institute of Technology, Karlsruhe, Germany

On January 30, 2020, the World Health Organization (WHO) declared the coronavirus disease 2019 (COVID-19) pandemic a Public Health Emergency of International Concern (PHEIC). On May 5, 2023, it declared that COVID-19 no longer constituted a PHEIC. Although COVID-19 has not been completely controlled or eradicated yet, it has been subsiding for more than a year and the world has returned to normal life.

## SIGNIFICANCE OF 1/23

**Figure 1:**
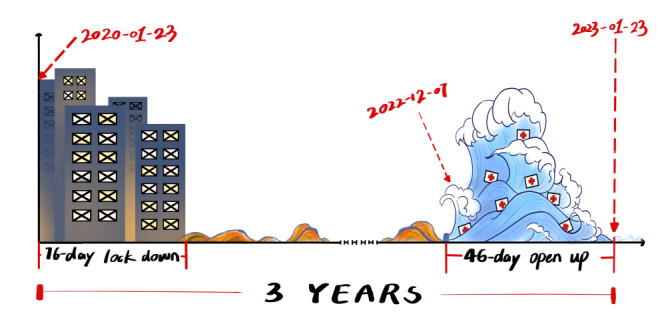
China’s three-year battle against COVID-19.

The birth and death date of the PHEIC status are arbitrary since they are decided by the meeting schedules of the relevant WHO committee. Here, we argue that if a single date should be remembered in the long-fought global battle against COVID-19, it should be January 23. On January 23, 2020, China began to lock down a city of more than 10 million inhabitants for seventy-six days [1]. This has never been done before, not even during the two World Wars. On January 23, 2023, forty-six days after China adjusted its COVID-19 control strategy that led to approximately one billion cases of infection, it was finally able to “flatten the curve” [2]. This was likewise an unprecedented achievement, not seen in any other pandemic in human history. These two dates mark the beginning and the ending of China’s three-year battle against COVID-19 (**Figure 1**).

It is a common practice across all cultures, that certain dates are marked to denote historical events or to reflect social fashion. For example, “9/11” is for an unprecedented terrorist attack day in the USA, while “11/11” is an unofficial Singles’ Day in China because the numeral 1 resembles a bare stick and therefore an unmarried man. For the historic COVID-19 pandemic that adversely affected many individuals and caused millions of deaths, we propose January 23 (shortened to “1/23”) as the date for the international scientific community to research and reflect upon.

## COVID-19 CONTROL IN CHINA

### The beginning

On January 23, 2020, unprecedented pandemic control measures were enacted in China: Wuhan, a city with more than 10 million residents, was locked down for seventy-six consecutive days [1], allowing for successful management of the transmission and securing precious time for China and the international community to tackle the pandemic. The impact of this lockdown on slowing down the COVID-19 pandemic was comprehensively evaluated in previous studies [3,4]. Never before had such a large city experienced such a long lockdown. China chose not to take chances, unlike other countries which took what was later called “Sweden’s Gamble” approach in managing the pandemic [5]. If there are still critiques of the lack of actions in the early days of COVID-19 control in China, we urge them to consider what occurred on January 23, 2020, in China and whether this could be ever done in any other country if a pandemic on the scale of COVID-19 scale struck again.

### The turning point

Since the initial Wuhan lockdown, countries worldwide adopted similar practices. However, the COVID-19 landscape changed significantly in 2022, when the highly transmissive and low pathogenic Omicron variant emerged [6]. Simultaneously, prolonged lockdowns in megacities caused major disruptions of economy, trade, and commerce and adverse social, psychological and health effects [7]. Considering the changed cost-benefit trade-off, China adjusted its strict COVID-19 strategies, notably by announcing “10 measures” on December 7, 2022 [8], halting all zero-COVID-19 practices and consequently leading to a surge of COVID-19 cases. Up to 90% of the Chinese population got infected within less than a month [9], a threshold indicating herd immunity for the Omicron variant. For Beijing, the capital city of China, it was projected that the cumulative infection attack rate was 75.7% on December 22, 2022, half a month after the “opening-up” [2].

### The ending

During most of December 2022 and January 2023, hospitals and community health centres across China faced many difficulties, reflecting the multi-faceted standards of China's health system. Death and suffering can never be taken lightly. However, on January 23, 2023, all major metrics on COVID-19, including positive cases and clinic visits, reached their lowest point. This was a defining moment in China’s fight against COVID-19. In less than two months, efforts to “flatten the curve” showed drastic success [10]. The most populous country in the world started to breathe and travel freely again, right before its most celebrated Lunar New Year of 2023. Soon after, countries which enacted traveling and testing restrictions for visitors from China during early 2023 gradually took them down.

## RESEARCHABLE ISSUES

### Case tally issue

There had been doubts that China might have under-reported the number of COVID-19 cases. However, these issues have been observed in many countries throughout the pandemic – for example, with the dubious “no testing, no cases” experience in the USA, or the scientific struggle to understand why this pandemic seemingly spared Africa [11]. For China, there are at least two reasonable responses to argue against this suspicion. First, the country’s health authorities projected that up to 90% of the Chinese population got infected within less than a month [9]. Realistically, it is not likely that the true number of infected cases was much higher than those projections. Second, keeping an accurate, mathematical tally of cases without life-threatening symptoms was not a priority for a country that, at the time, struggled to save the lives of those in danger. Instead of offering suspicion, the international community should offer solutions, including a globally accepted network and gold standards on how to survey and report cases.

### Death tally issue

Some also questioned that China might have underreported the number of COVID-19 deaths. Before the policy shift in December 2022, the international research community had projected that more than one million people would die if China completely gave up pandemic control measures [12]. We do not intend to argue for or against the accuracy of this projection, since we do not have enough data. However, there are wide variations in excess death estimates worldwide; the global excess death toll could be close to 15 million, while only around five million were reported as being caused by COVID-19 [13]. Furthermore, there is still no consensus on the difference between “death from COVID-19” and “death with COVID-19” in the research community. For example, a completely healthy person killed in a car accident will not be counted as a COVID-19 fatality simply because he/she was also COVID-19 positive without demonstrating any symptoms or adverse health effects. Again, the international community needs to provide a globally accepted network and gold standards so that statistics on future pandemics are uniform and comparable.

### Timing issue

The timing of China's strategy shift in December 2022 was also debated because other respiratory infectious diseases, including influenza, intensify in transmission during winter; others worried that the country was not yet ready for removing measures, not at least due to the relatively low vaccination rate, especially for the booster dose. Others were concerned about the huge population movement right before China’s lunar New Year’s Day (January 22, 2023). Considering the changed nature of the pandemic and economic pressures, China felt the need for immediate action. The pandemic needed to run its natural course and action needed to be taken “right away”. There is a famous saying in China: “A long pain is worse than a short pain”. China took the short pain – although the route was intensely painful, the population and the nation faced the challenge and swiftly won the battle.

## REFLECTION AND CONCLUSION

The international community recognised COVID-19 as a significant health emergency. The world can learn valuable lessons from China's response to the pandemic, especially their actions on January 23 of 2020 and 2023 [14]. These two dates are defining moments for dealing with a global pandemic crisis. What was written in textbooks could be too dogmatic, but impractical. What was taught in classrooms could be too ideological, yet inexecutable. As the world admires the human challenge experiments, in which three dozen volunteers in the United Kingdom participated [15], it should also reflect and learn from a full-scale nation challenge experiment that occurred in China. It provides valuable study material for current and future public health professionals, as well as painful lessons for both the East and the West. The sacrifices made and the capacities built by the international communities must not go to waste. What we learned from COVID-19 controls that occurred on January 23 of 2020 and 2023 should be transformed into meaningful and lasting global health knowledge.
